# Gels in Heterogeneous Photocatalysis: Past, Present, and Future

**DOI:** 10.3390/gels10120810

**Published:** 2024-12-09

**Authors:** Fitri Rizki Amalia, Lei Wang, Zuzanna Bielan, Agata Markowska-Szczupak, Zhishun Wei, Ewa Kowalska

**Affiliations:** 1 Faculty of Chemistry, Jagiellonian University, 30-387 Kraków, Poland; fitri.rizki.amalia@uj.edu.pl (F.R.A.); lei.wang@uj.edu.pl (L.W.); 2 Department of Chemical and Process Engineering, West Pomeranian University of Technology in Szczecin, 71-065 Szczecin, Poland; agata.markowska@zut.edu.pl; 3 Hubei Provincial Key Laboratory of Green Materials for Light Industry, New Materials and Green Manufacturing Talent Introduction and Innovation Demonstration Base, Hubei University of Technology, Wuhan 430068, China; wei.zhishun@hbut.edu.cn

**Keywords:** aerogels, cryogels, encapsulation, floating titania, gel photocatalysts, heterogeneous photocatalysis, hydrogels, microrobots, recycling, xerogels

## Abstract

Photocatalysis has attracted more and more attention as a possible solution to environmental, water, and energy crises. Although some photocatalytic materials have already proven to perform well, there are still some problems that should be solved for the broad commercialization of photocatalysis-based technologies. Among them, cheap and easy recycling, as well as stability issues, should be addressed. Accordingly, the application of gels, either as a photocatalytic material or as its support, might be a good solution. In this review, various propositions of gel-based photocatalysts have been presented and discussed. Moreover, an easy nanoarchitecture design of gel-based structures enables fundamental studies, e.g., on mechanism clarifications. It might be concluded that gels with their unique properties, i.e., low density, high specific surface area, great porosity, and low-cost preparation, are highly prospective for solar-energy-based reactions, water treatment, photodynamic cancer therapies, and fundamental research.

## 1. Introduction

Heterogeneous photocatalysis has been considered an excellent method for solar energy conversion into valuable fuels as well as the purification of water, air, and surfaces [1,2,3,4]. It is believed that the most wanted processes driven by solar photocatalysis, i.e., overall water splitting (into hydrogen and oxygen) and artificial photosynthesis (reduction of carbon dioxide with simultaneous evolution of oxygen), could solve humanity’s top problems, such as environmental pollution, global warming, energy crisis, etc. [5,6,7].

Titanium(IV) oxide (titania) is probably the most widely investigated semiconductor photocatalyst due to its many advantages, i.e., high activity, stability (photo-, thermal-, and chemical-), negligible toxicity, low price, and abundance [8,9,10,11]. However, a high level of photocatalytic activity usually means only a UV response (inactivity under visible light (vis) range of solar radiation) because the most active materials are characterized by a wide bandgap of ca. 3.0 eV. Accordingly, either UV light must be applied, and thus low activity is observed under natural solar radiation consisting of only ca. 3% UV, or semiconductors with narrower bandgaps must be used, but then, the rate of charge carriers’ recombination is fast, and often their redox performance is not sufficient to drive specific reactions [12]. Therefore, various approaches have been proposed to achieve high photocatalytic activity under natural solar radiation, such as doping, surface modification, heterostructure formation, and nanoarchitecture design [13,14,15,16,17]. Although the formation of heterostructures, based on the Z-/S-scheme mechanism, is probably the most efficient for achieving highly active and stable photocatalysts [18,19,20], the morphology of semiconductors also influences photocatalytic performance. In some cases, a significant increase in activity has been observed by the formation of a specific morphology [21,22,23,24,25,26]. Moreover, nanoarchitecture design might also help to clarify the mechanism of photocatalytic reactions [20,25,26,27,28]. For example, very interesting findings on the mechanism of plasmonic photocatalysis have been presented based on two types of titania aerogels modified with gold nanoparticles (NPs) [29]. Accordingly, the structure of the aerogel allows the discussion of the mechanism (as discussed in the next section).

Nowadays, gels are used in various applications, such as for sensors, energy storage, and environmental purification [30,31,32,33], but their use in photocatalysis is not as common. However, more and more studies have recently been performed with gels either as a photocatalyst itself or an inert/active support for photocatalysts due to their various advantages, such as low density, large specific surface area, great porosity, and low costs [34]. Because of those properties, the utilization of gels in photocatalysis is very promising. It is thought that efficient light harvesting and easy separation of photocatalysts from reaction systems (usually in suspension), which are often the main concerns in photocatalysis, could be solved by the application of gels. Although there are many interesting reports on gels in photocatalysis, including also some review papers [35,36,37,38], most of them focus only on one material, e.g., silica-based gels or titania aerogels. Here, we are trying to show “a big picture” of the recent progress of gels in photocatalysis by discussing various studies about gels made from different materials, which is very important for the development of new materials.

Accordingly, it is worth investigating how gels could be efficiently used in heterogenous photocatalysis and how/if the properties of gels might control the photocatalytic performance. Therefore, the reported studies on gel-based photocatalysts have been summarized and discussed in this paper, showing their advantages and disadvantages, as well as the future directions on the development of photocatalytic gels and relevant topics.

## 2. Gels

**Figure 1:**
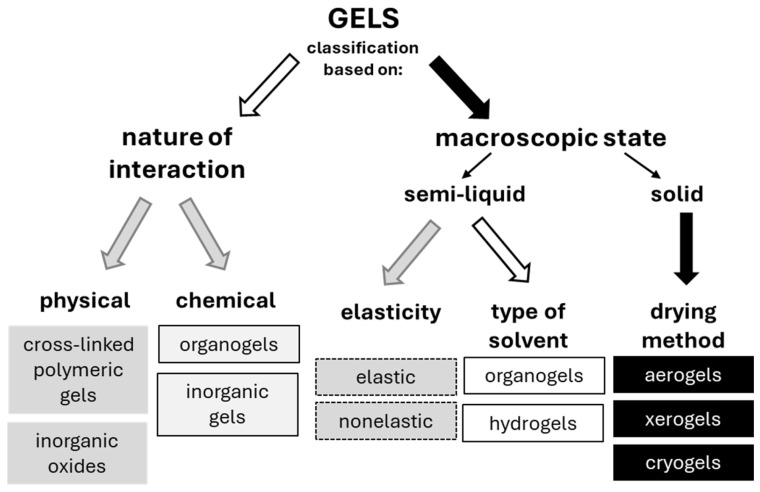
Schematic drawing of gels’ classification (drawn, based on Reference [39]).

The large group of gels can be divided into respective subgroups, considering several different parameters, such as the following:

(i) Preparation method;
(ii) Nature of origin: natural (proteins, polysaccharides), semi-synthetic (cellulose derivatives), and synthetic polymers;
(iii) Nature of bonds involved in the three-dimensional (3D) network, i.e., (a) dispersed solids with Vander Waals or electrostatic interactions and (b) hydrophilic polymers of two types: type I (covalent bonds between macromolecules) and type II (hydrogen bonds between polymers);
(iv) Physical and chemical characterization;
(v) Macroscopic state (continuous phase): (a) semi-liquid (organogels and hydrogels) and (b) solid gels (aerogels, xerogels, and cryogels), as shown in the right part of Figure 1.

In the case of organogels and hydrogels, the dispersed phase is liquid, i.e., organic solvent and water, respectively. The high content of liquid (even up to 90%) causes a semi-liquid (also called “semi-solid”) consistency, perfect for medicine applications like medical dressings, drug-delivery systems, scaffolds in tissue engineering, environment sensitivity detectors, contact lenses, ECG medical electrodes, glue, etc. [40]. They are also frequently used for environmental applications, e.g., for oil/water separation. Unfortunately, they are characterized by poor thermal and environmental stability, and thus, they are almost useless in photocatalytic processes, except as a support for photocatalysts. In contrast, solid gels containing air as a dispersed phase are 3D materials with well-developed specific surface areas and high porosity. Moreover, their stability to environmental conditions is much higher than that in semi-liquid gels [41,42]; thus, they could be used as a promising new group of photocatalysts for water/air purification and energy conversion (water splitting, photocurrent generation, artificial photosynthesis).

There are three types of solid gels, i.e., xerogels, cryogels, and aerogels, which differ in drying methods used during their formation. Accordingly, xerogels are formed by evaporating water by air-drying during a temperature increase or a pressure decrease [39]. Xerogels are characterized by fine pores (1–10 nm) and a large specific surface area of 100–900 m^2^ g^−1^ [43]. The high level of shrinkage (>90%), caused by high capillary pressure in the wet gel, results in the destruction of the initial uniform form of the gel [39].

In the case of cryogels, drying of natural or synthetic polymers is carried out by lyophilization (freeze drying), i.e., the solvent inside the gel is first frozen and then sublimated, and thus shrinking of the 3D gel structure is completely avoided. However, many factors (e.g., the amount of water and organic co-solvents, ionic strength, pH value, gel composition, and conditions of freezing, such as cooling steps, cooling rate, and temperature gradients) might influence the final properties of the cryogels (pore size, wall thickness, and other structural properties) [39].

The third group of solid gels—aerogels—is considered the most interesting, named “a new form of matter” and “a superior material” with a very high specific surface area of 500–1000 m^2^ g^−1^, with low sound transmission and exceptional thermal insulation [39]. Aerogels are formed by the removal of the solvent under control conditions to maintain the 3D structure (e.g., high-temperature and low-temperature supercritical fluid drying (SCFD)), and thus with a low level of shrinkage (<5%) [44,45,46]. The International Union of Pure and Applied Chemistry (IUPAC) defines aerogels in a very general way as a low-density, ultralight, microporous solid material formed from a gel, where the dispersed liquid is replaced by a gas [47]. The group of aerogel materials, initially obtained by removing water from jellies, was later extended by silica and alumina [48] up to the final inclusion of transition metal oxides, like ZrO_2_, NiO, and TiO_2_ [49]. Although the most typical aerogels are still silica-, organic- and carbon-based ones [50], semiconductor aerogels, especially metal oxides, have been more and more popular in recent years. Metal oxide aerogels have attracted the most attention in the field of photocatalysis. Using their unique properties, like high specific surface area [51], high porosity [52], and semiconductor nature, they have become a new class of active photocatalytic materials, as discussed further.

## 3. Gels in Photocatalysis

There are two main functions of gels in photocatalysis, i.e., either as a photocatalyst itself (mostly in the form of solid gel) or as a support for a photocatalyst. Aerogels and cryogels (often named aerogels) are the most common types of gels used in photocatalysis, whereas considering the chemical composition, the same as in the case of other studies on heterogeneous photocatalysis, titania has been used/investigated the most. Accordingly, titania aerogels have been prepared, characterized, tested, and discussed in many studies. Therefore, in this review, titania aerogels are presented first (Section 3.1), then other chemical compounds in the form of gels used as photocatalysts are presented (Section 3.2), and finally, gels as support for photocatalysts and for other applications are discussed (Section 3.3).

### 3.1. Titania Photocatalysts in the Form of Solid Gels

**Figure 2:**
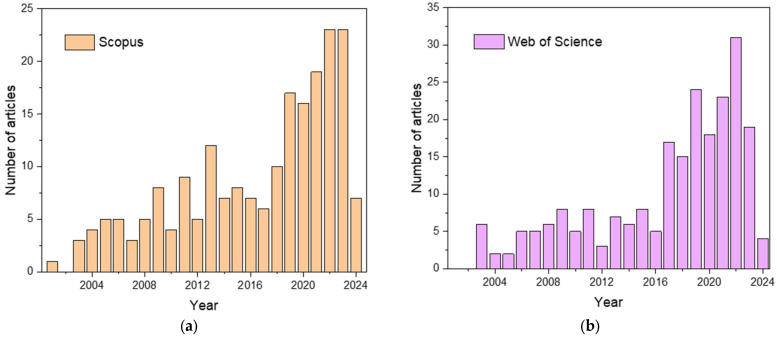
Evolution of the number of publications about titania aerogels used in photocatalysis, according to (**a**) Scopus and (**b**) Web of Science databases. Access on 21 September 2024; used query: “Titania OR TiO_2_ AND Aerogel AND Photocatalysis”.

Titania is a photocatalyst investigated the most since the famous work by Fujishima and Honda [53]; hence, the photoactive titania aerogels are obviously of great interest [36]. The first papers on this topic were published at the beginning of the XXI century, and since then, their number has been increasing year by year (see Figure 2).

In this review, the preparation of titania aerogels (with their performance) is presented first, then modified titania aerogels are discussed, and finally, the stability issue is considered.

#### 3.1.1. Preparation, Properties, and Performance of Pristine Titania Solid Gels

**Figure 3:**
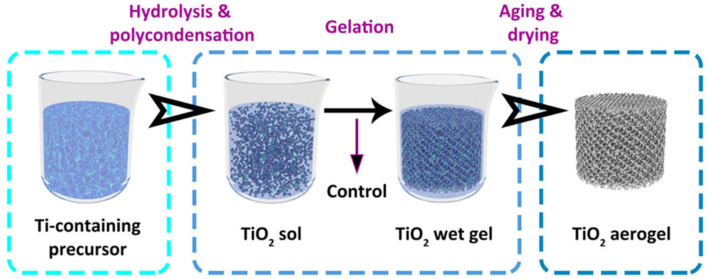
Sol-gel synthesis of titania solid gels. Reprinted with permission from Reference [56]. Copyright (2023) Creative Commons Attribution.

The most common and at the same “classical” method of titania preparation is a sol-gel process, in which titania is synthesized first from its precursor (mostly titanium alkoxides via hydrolysis), then precipitated (from an aqueous solution), washed, and finally, dried in an air/vacuum/inert atmosphere, as presented in Figure 3. Materials obtained in this process are usually dried via normal thermal treatment in an oven, and thus, xerogels are mostly formed. Accordingly, during solvent removal, surface tension inside pores causes their partial collapse and, consequently, deterioration of the material’s texture and surface properties. Two different solutions are usually proposed to avoid structure shrinkage and, thus, the destruction of the 3D gel network. The first one is based on the use of solvents with low surface tension or the addition of surfactants, which lower the surface tension of the solvent [54]. In the latter, the most efficient and commonly used method (especially on a laboratory scale) is where solvents are removed under supercritical conditions [55], which results in the formation of titania aerogels with the preserved gel skeleton [56].

The research by Dagan and Tomkiewicz [57,58] has been considered a pioneering study on sol-gel preparation of titania solid cryogels (solvent removal by freeze drying) and aerogels (supercritical drying). Interestingly, though the drying method was different, aerogels exhibited better photocatalytic properties for salicylic acid removal than analogically prepared cryogels [58], which was even three times higher than that by commercial P25 (a famous titania photocatalyst with usually the highest photocatalytic activity among various titania materials for both oxidation and reduction reactions [59,60,61,62]). It was proposed that better photocatalytic activity of aerogels was caused by larger specific surface area and greater porosity than that in xerogels/cryogels (obtained by an analogical procedure), and thus better adsorption capacity of pollutants/reagents.

**Figure 4:**
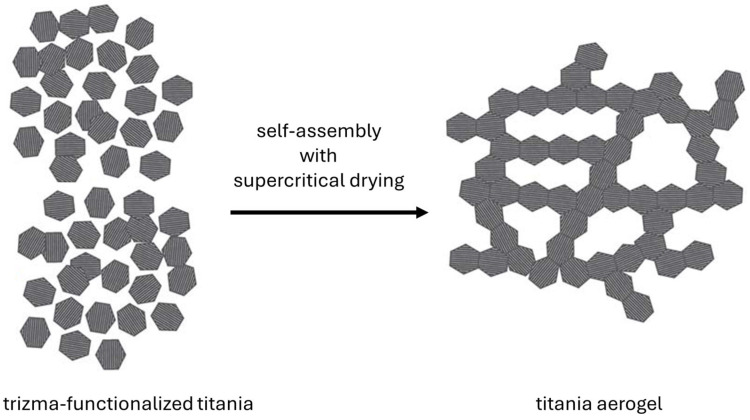
The scheme of titania aerogel formation using crystalline nanoparticles’ self-assembly method (drawn based on Reference [67]).

It should be pointed out that during the preparation of aerogels by a sol-gel method, titania is synthesized first from its precursor (usually alkoxides), and, thus, the obtained material is amorphous. Accordingly, additional post-treatment operations (usually heating) are needed to crystallize titania (as an amorphous form exhibits low photocatalytic activity due to a large number of electron traps working as a recombination center of charge carriers [63,64,65,66]), which could cause the destruction of aerogel structure. In order to solve this problem, another method of aerogel synthesis has been proposed, based on the self-assembly of crystalline titania nanoparticles (NPs), resulting in the formation of long 3D chains of high porosity (see Figure 4). The spontaneous connection of particles is caused by pre-functionalization of the titania surface [67]. A variation of this method was described by Luna et al., in which titania functionalized by trizma (2-amino-2-(hydroxymethyl)-1,3propanediol) was obtained first by a sol-gel process in a non-aqueous medium [68]. In the next step, noble metals (Au and/or Pd) were deposited on the titania surface. In the final and most important step, the metal-loaded titania was irradiated with UV light to initiate the gelation process. The obtained PdAu–titania aerogels exhibited nano-sized properties despite their macroscopic dimensions, which resulted in high photocatalytic activity for hydrogen generation under UV light.

Another interesting approach has been proposed by Dilger et al. for the synthesis of titania aerogels in a gas phase using a specially designed three-chamber oven [69]. In the first step, a liquid precursor of titania is introduced to the evaporation zone, in which its complete vaporization proceeds. Further, in the particle formation zone (the second chamber), the nucleation and growth of titania particles take place. The last step occurs in the sequestration zone, where, due to the temperature difference between the interior of the hot oven and the cold walls, the aerogel resublimes.

Compared to the sol-gel and the self-assembly methods, titania aerogels obtained in a gas phase are usually characterized by higher purity and crystallinity due to solvent-free synthesis [56]. However, the gas-phase aerogel synthesis is far more difficult to control since a lot of process parameters must be optimized simultaneously (the temperature in three zones, inject gas flows, aerosol concentration, etc.). Accordingly, this method is rarely used for aerogel synthesis nowadays. Nevertheless, it is successfully applied for preparation of faceted structures, such as decahedral anatase particles (DAP) [21,22,70] and CH_3_NH_3_PbBr_3_ perovskite microcrystals [71]. The summarized comparison between the three methods of titania aerogels’ preparation is shown in Table 1, whereas exemplary data of photocatalytic activity are presented in Table 2 (although it should be mentioned that photocatalytic activity data are hardly comparable because of significant differences in experimental methods). Considering photocatalytic performance, all samples exhibit very high photocatalytic activity (comparable to that of P25—the common standard used for activity comparison), and the most important is their form of application—a solid gel, which allows a fast and cheap recovery of the photocatalyst after reaction. Unfortunately, no data on the photocatalytic activity of samples prepared via a gas-phase method could be found in the literature. To conclude, it is thought that samples prepared by a sol-gel method are the cheapest (including even costly supercritical drying and calcination processes), and the whole procedure is easy to control. Therefore, this synthesis method is the most advisable, especially for the “first-time users”, i.e., researchers without experience in self-assembly-based methods.

#### 3.1.2. Preparation, Properties, and Performance of Titania–Silica Solid Gels

It should be mentioned that the first aerogels used for photocatalysis were in the form of titania–silica composites (see Table 3). Compared to titania, silica aerogels are characterized by an even larger specific surface area and greater porosity [20], which allows better light-harvesting ability and pollutant adsorption. It is also worth mentioning that those composites often reveal higher photocatalytic activity than pure titania aerogels despite the lower content of titania in the product [56,74]. A great example is the work of Kim et al. [75], in which the photocatalytic activity of titania–silica aerogels with different ratios of Si-to-Ti was compared to that of P25. During 70 min UV irradiation, Methylene Blue (MB) dye was almost completely decolorized on the composite with a Ti-to-Si ratio of 0.43, reaching the reaction rate constant (K) of 0.0685 min^−1^, which was almost double that of P25 (0.0356 min^−1^). Similarly, Gan et al. tested different molar ratios of titania to silica, as well as different calcination temperatures of TiO_2_/SiO_2_ aerogels for pyridine photodegradation, and it was found that almost complete removal of pyridine from an aqueous phase could be achieved within four hours of UV irradiation [76].

#### 3.1.3. Preparation, Properties, and Performance of Titania Solid Gels Modified with Metals

Although titania and titania–silica aerogels exhibit high photocatalytic activity, they are only active under UV irradiation because of their wide-bandgap values. Accordingly, to utilize solar radiation efficiently, further modifications are needed. Among various methods, such as composite formations with other semiconductors like reduced graphene oxide (rGO) [80], graphitic carbon nitride (g-C_3_N_4_) [81], and metal–organic frameworks (MOF) [82], the surface modification with metals is still the most popular. By combining a large surface area and a high porosity of titania aerogels with localized surface plasmon resonance (LSPR) of noble metals, such as platinum, gold, silver, and copper, the resultant materials exhibit significant photocatalytic activity towards the decomposition of various pollutants (see Table 4), and generation of hydrogen by water splitting [29,68,83,84]. The study by DeSario et al. on Cu/TiO_2_ aerogel deserves special attention [85], in which the elimination of sarin (a highly toxic organophosphorus compound used as a mass destruction weapon during WWII) from a gas phase has been successfully performed under vis irradiation (λ > 480 nm).

**Figure 5:**
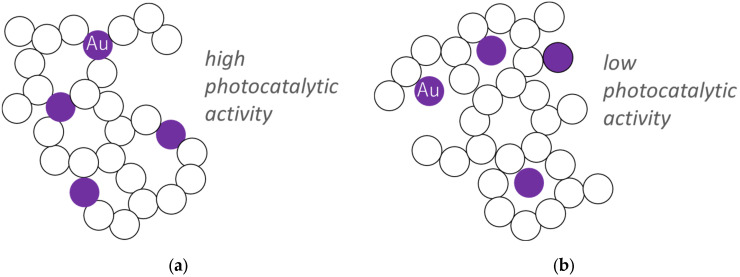
The schematic drawings of titania aerogels with gold NPs (violet balls): (**a**) incorporated inside the 3D network (3D Au-TiO_2_); and (**b**) in porosity (DP Au-TiO_2_) (drawn based on References [29,86]).

The most interesting aspect of research with aerogels is based on the morphology-based possibility of mechanism investigations. For example, the importance of the titania–gold interface has been attested for the 3D network of titania aerogels with two kinds of morphology, i.e., with gold NPs (diameter of ca. 5 nm) either incorporated inside the network (3D Au-TiO_2_, i.e., gold NPs replacing some titania NPs) or deposited in the porosity (DP Au/TiO_2_), as shown in Figure 5 [29,86]. Interestingly, the 3D Au-TiO_2_ structure exhibits much higher photocatalytic activity under vis irradiation than the respective DP Au/TiO_2_ one, despite very similar properties (same size and content of gold NPs and same features of titania aerogel). Accordingly, it has been concluded that the interface between titania and gold is crucial for plasmonic photocatalysis. Although two possible mechanisms have been suggested, i.e., charge transfer (“hot” electron transfer from gold to the conduction band of titania) and energy transfer, it is expected that enlarged contact between gold and titania might accelerate the direct transfer of charges. Interestingly, further research on plasmonic photocatalysis has also confirmed that the interface between semiconductor and noble metal is a key factor of vis activity, e.g., highly active TiO_2_(a)-Au-TiO_2_(b) samples, in which gold NPs deposited on titania of large particles (TiO_2_(a)) were additionally partly covered by fine particles of another titania sample (TiO_2_(b)) [87].

**Table 4:** Summary of the selected metal-modified TiO_2_ aerogel photocatalysts.

Sample	Synthesis Method	BET * /m^2^ g^−1^	Photocatalytic Activity Tests			Ref.
			Irradiation	System	Effect *	
PdAu-TiO_2_	Self-assembly with gelation induced by light	450	sim. solar	H_2_O/MeOH ^†^	22 mmol·g^−1^·h^−1^ (H_2_)	[68]
Pt/TiO_2_	Sol-gel with supercritical drying	85	UV/vis	H_2_O/MeOH ^†^	328 mmol·g^−1^·h^−1^ (H_2_)	[83]
3D networked Au-TiO_2_	Sol-gel with supercritical drying	144	vis	PEC H_2_O ^††^	0.4 IPCE	[29]
Pd/TiO_2_	Microwave with further solvent-exchange and supercritical drying	423	vis	H_2_O/MeOH ^†^	32.8 mmol·g^−1^·h^−1^ (H_2_)	[84]
Cu/TiO_2_	Sol-gel with supercritical drying	153	vis	d. sarin	degradation enhanced	[85]
Ni-, Co-, Cu-, Fe-doped TiO_2_	Sol-gel with supercritical drying	129 (Ni) 116 (Co) 158 (Cu) 158 (Fe)	UV/vis	d. AO7	K_Ni_: 2.0·10^−3^ min^−1^ K_Co_: 6.0·10^−4^ min^−1^ K_Cu_: 1.6·10^−3^ min^−1^ K_Fe_: 7.0·10^−4^ min^−1^	[35]
Ag/N-TiO_2_ paper	Sol-gel	447	UV	d. MB d. HCHO	~100%, 240 min ~55%, 720 min	[88]

MeOH—methanol, d.—degradation, IPCE—incident photon-to-electron conversion efficiency, AO7—Acid Orange 7, sim. solar—simulated solar light, K—reaction rate constant, * rounded to the nearest integer (when possible), ^†^ water splitting with/without a sacrificial agent, ^††^ photoelectrochemical (PEC) water splitting.

**Figure 6:**
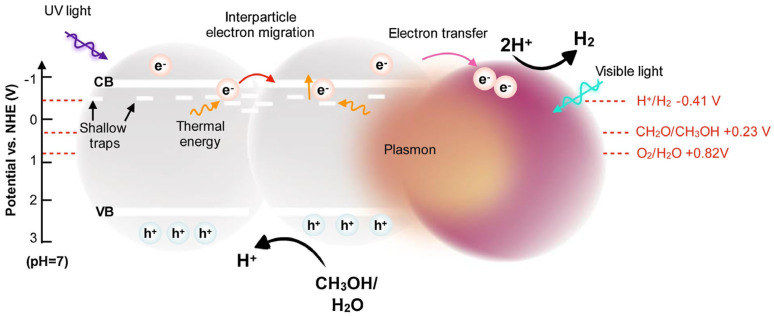
The scheme showing hydrogen evolution under solar irradiation on titania aerogels with noble metals’ NPs (violet balls) incorporated inside the 3D network. Reprinted with permission from Reference [68]. Copyright (2020) Elsevier.

Another interesting study on plasmonic photocatalysis has been performed by Luna et al. for titania aerogels with incorporated noble metals inside the 3D network, similar to the work discussed above (the left part of Figure 5), but for mono- and bi-metallic composites, i.e., Pd, Au, and PdAu [68]. In this study, the time-resolved microwave conductivity (TRMC) method was applied for the analysis of the charge carriers’ mobility and lifetime. It has been confirmed that under UV irradiation, noble metals work as an electron pool, inhibiting the recombination of charge carriers. In contrast, under vis irradiation, the clarification of the mechanism is more complex. It has been proposed that inactivity at 530 nm and activity at 430 nm suggest that hydrogen evolution proceeds via an energy transfer mechanism, which is reasonable considering that plasmon resonance of gold (usually at ca. 520–550 nm for spheric NPs) could not match with titania photoabsorption. However, it should be pointed out that the distinction between the different functions of noble metals in photocatalysis, i.e., as an electron scavenger, a catalytic site, a plasmonic sensitizer, but also an “inner filter” (lowering photocatalytic activity due to a shielding effect), is challenging, and in many cases almost impossible, especially for the hydrogen evolution reaction under UV/vis irradiation [89,90,91]. The most interesting finding of the study by Luna et al. focuses on the role of aerogels in activity enhancement. It has been proposed that aerogel structure might facilitate the migration of photogenerated electrons via shallow electron traps between interconnected titania NPs, resulting in highly efficient interparticle electron transfer, as shown in Figure 6, similar to the enhanced activity by faceted titania [92].

#### 3.1.4. Stability of Titania Solid Gels

**Figure 7:**
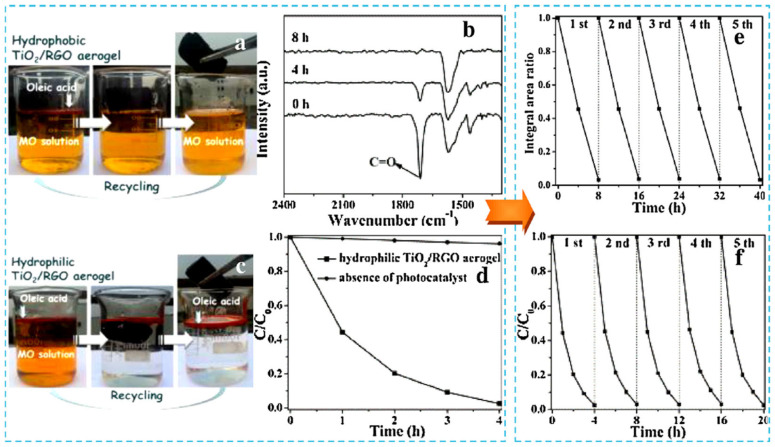
The experimental data showing (**a**,**b**) the color changes during removal of dyes using hydrophobic (**a**) and hydrophilic (**b**) titania/rGO aerogels; (**c**) FT-IR spectra showing oleic acid photodegradation over time in the presence of hydrophobic titania/rGO aerogel; (**d**) comparison between photolysis and photocatalysis during MO removal using hydrophilic titania/rGO aerogel; recycling experiments during (**e**) oleic acid and (**f**) Methyl Orange removal. Reprinted with permission from Reference [95]. Copyright (2015) Elsevier.

Nowadays, the stability of photocatalysts is almost as important as their photocatalytic activity. The possibility of semiconductors’ reusing in more than one process is in agreement with the 5 R’s method (“refuse, reduce, reuse, repurpose, and recycle”) and sustainability in a broad sense. Nevertheless, surprisingly, only a little attention is devoted to the stability and reuse of titania aerogels in recent literature. For example, Ferreira-Neto et al. tested the stability of SiO_2_/TiO_2_ aerogels, considering only the resistance to high temperature, more precisely, if the material could keep its properties after calcination at 1000 °C [93]. However, no reusability tests were conducted during tests of photocatalytic activity (photodegradation of Rhodamine B (RhB). Work by Zhang et al. is one of the few exceptions [94]. Their cellulose nanofiber/titania/chitosan (CTC) aerogel composite has remained photoactive even after seven consequent cycles of tetracycline degradation under UV light, with only less than 10% loss in activity. Moreover, detailed characteristics of the material were performed after the last cycle, and it was confirmed that the properties of re-used materials were comparable with fresh, just prepared ones. In turn, Liu et al. developed titania/rGO aerogel composites of changeable surface wettability (depending on the amount of titania used) [95]. Such composites showed selectivity towards photo-decomposed compounds (Methyl Orange (MO) and oleic acid layered with Sudan III dye for better visualization of changes). Apart from high photocatalytic activity, the composites were characterized by significant stability up to five photocatalytic cycles, which is presented in Figure 7.

### 3.2. Other Photocatalysts in the Form of Gels

Although titania aerogels are the most popular, attracting considerable attention due to high photocatalytic activity and stability (typical for all titania materials), other chemical compounds, especially those with narrower bandgap than that in titania (for efficient use of solar energy), such as CdS, CdSe, MoS_2_, and C_3_N_4_, have also been prepared in the form of gels, and tested in various photocatalytic reactions, as discussed in this section.

#### 3.2.1. Single-Component Gels

Like the preparation of titania aerogels, the sol-gel method followed by drying is commonly used for the synthesis of other solid gels. For example, CdS aerogels could be synthesized by the sol-gel method from CdO via CdS nanocrystals (NCs)’ formation, gelation (oxidation of thiolate surface ligands), and subsequent supercritical CO_2_ drying [96]. It has been found that CdS aerogels exhibit much higher photocatalytic activity than original CdS NCs for degradation of dyes (MB and MO) under vis irradiation, possibly due to an efficient charge carriers’ separation and a high specific surface area. Moreover, additional annealing of CdS aerogel results in a further increase in photocatalytic activity as “a consequence of strengthening the connectivity between NCs in the porous network and removing the surface ligands”. However, it should be pointed out that dyes should not be used as model molecules for vis activity testing due to the sensitization mechanism, as also clearly observed in this report by the shift of the MB absorption peak during irradiation, caused by de-methylation of MB [97,98,99,100].

**Figure 8:**
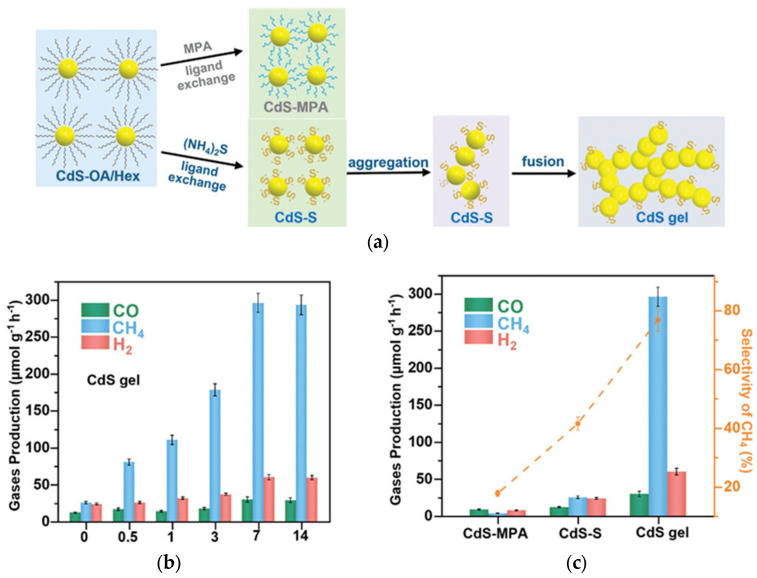
(**a**) The schematic drawing of CdS QD gel formation; (**b**,**c**) the photocatalytic activity for CO_2_ reduction: (**b**) the activity during different aging durations (days presented on the x-axis); and (**c**) the activity comparison between CdS QD gel and reference samples. Reprinted with permission from Reference [101]. Copyright (2024) Wiley.

Similarly, CdS quantum dot (QD) gels have been proposed for efficient separation of charge carriers by Xu et al., but in the form of semi-liquid/solid gels [101]. In this study, CdS QD gels have been synthesized through the assembly of S-capped CdS (CdS-S). First, oleic acid-capped CdS QDs (CdS-OA) were treated separately with mercaptopropionic acid (MPA) and (NH_4_)_2_ to replace oleic acid ligands, resulting in the formation of MPA-capped CdS QDs (CdS-MPA) and S-capped CdS (CdS-S), respectively. The aggregation of QDs was more efficient in the case of CdS-S QDs than CdS-MPA because of shorter S^2−^ ligands and, thus, better contact between QDs. Then, resultant CdS QD gels were obtained by 7-day aging, as shown in Figure 8a. On the contrary, CdS-MPA QDs did not form a gel structure, remaining well-dispersed after identical treatment. Interestingly, it has been found that aging duration is critical for photocatalytic performance (CO_2_ reduction under vis), and 7 days is a necessary time to achieve the highest activity, resulting from complete gelation (Figure 8b). Obviously, CdS QD gel exhibits much higher photocatalytic activity than dispersed QDs (Figure 8c), possibly due to an efficient charge migration via the 3D network (as proven by photoelectrochemical tests). Moreover, the stability of CdS gel has also been confirmed during four cycles, verifying their high potential as vis-responsive photocatalysts.

Gels composed of other QDs have also been investigated, such as CdSe aerogels prepared through the 3D assembly of QDs capped with inorganic ligands ((NH_4_)_2_S) using only water as a dispersion solvent [102]. Interestingly, it has been found that these aerogels exhibit much higher photocatalytic activity than the respective powder sample of CdSe QDs toward CO_2_ reduction, probably due to a self-supported porous structure, contributing to light harvesting and reagent capture, as well as a beneficial ligand-free surface for the direct contact between QDs and CO_2_.

**Figure 9:**
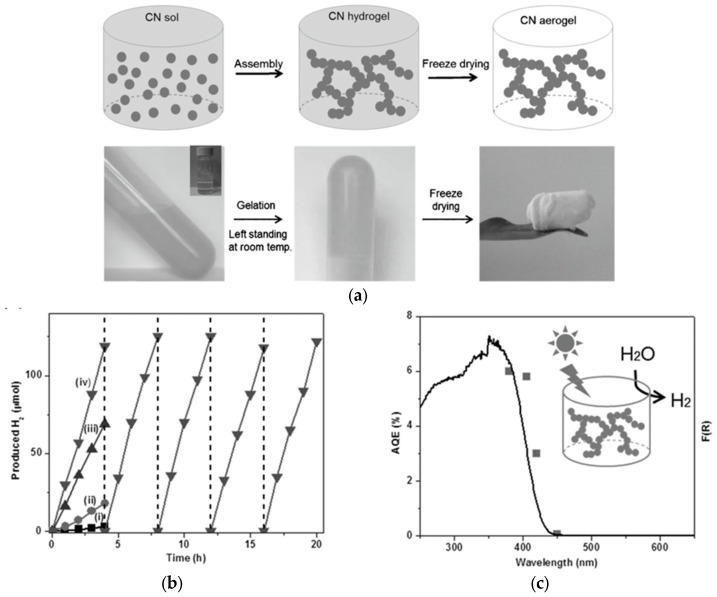
(**a**) The schematic drawing with respective photos of C_3_N_4_ cryogel fabrication; (**b**) Data of photocatalytic activity for hydrogen evolution under vis irradiation on C_3_N_4_ cryogel (sample (iv)) and reference samples ((i–iii) C_3_N_4_-based samples prepared analogously) modified with 3wt% of Pt as a co-catalyst; and (**c**) action (points) and absorption (line) spectra for H_2_ evolution on C_3_N_4_ cryogel modified with 3wt% of Pt as a co-catalyst. Reprinted with permission from Reference [106]. Copyright (2017) Willey.

Carbon nitride (C_3_N_4_) is another very popular material for vis-responsive photocatalysis, mostly due to its high reduction ability (e.g., reduction of water and carbon dioxide) and efficient light harvesting (potential to work under natural solar radiation) [103,104,105]. Obviously, C_3_N_4_ in the form of gels has also been proposed, synthesized, and tested. For example, Ou et al. have developed a self-assembly method to form C_3_N_4_ cryogels, as presented in Figure 9 [106]. It has been found that this sample is stable (five 4 h cycles; Figure 9b) and highly active for both water reduction and water oxidation (to H_2_O_2_) under vis irradiation. Moreover, the action spectrum of hydrogen evolution correlates well with the absorption spectrum of the photocatalyst (Figure 9c), validating that the vis response is indeed caused by the photocatalytic activity of C_3_N_4_.

Another method to obtain vis-responsive materials is their doping, including also self-doping, e.g., the formation of defects. However, it should be noted that defects might also work as centers for charge carriers’ recombination, and thus, doping might result in decreasing (instead of increasing) photocatalytic activity [64,66,107,108,109,110,111,112,113]. Zhao et al. prepared MoS_2_ photocatalyst with S defects in the form of an aerogel through chemical cross-linking of functional ultrathin [114]. Although vis activity was achieved, the experiments were performed for dye decolorization, and thus, the sensitization effect could not be excluded. Moreover, a slight decrease in activity (ca. 10%) was observed during ten 3 h cycles.

#### 3.2.2. Multi-Component Gels

Although single-component gels have exhibited photocatalytic activity even under vis irradiation (as discussed above), their overall performance should be improved for broad applications—it must be remembered that vis response usually means limited redox properties, i.e., these materials are highly efficient either for reduction or oxidation reactions. Moreover, for some reactions, co-catalysts are necessary for high photocatalytic activity, e.g., metallic deposits for hydrogen evolution (as already mentioned above, Figure 9b,c). Accordingly, composite systems are highly needed, such as metal-modified materials and S/Z-scheme heterojunctions.

The most typical and easiest modification method for any semiconductor is the deposition of metal particles on its surface or inside its structure. Obviously, in the case of gel photocatalysts, such modification is also very popular, as already mentioned in Section 3.1.3. An interesting example has been presented by Tang et al. on the construction of a metal/semiconductor (Au/CeO_2_) aerogel through an epoxide addition sol-gel method [115]. In this study, the size of gold NPs is controlled by the amount of mercaptosuccinic acid (MSA) used for the reduction of gold cations. The most interesting finding of this research is the influence of irradiation wavelengths and the gold presence on the selectivity of CO_2_ reduction, depending on the photocatalytic mechanism, i.e., excitation of either semiconductor (with a subsequent electron transfer to gold) or noble metal under UV and vis, respectively. It has been found that under UV irradiation, only CH_4_ is formed in the case of pristine CeO_2_; both CO and CH_4_ products are generated on the gold-modified one, indicating that gold works as a co-catalyst for CO formation. However, under vis irradiation, again, only CH_4_ is created on the Au/CeO_2_ aerogel. Therefore, the mechanism of plasmonic charge transfer is expected, i.e., the transfer of “hot” (plasmonic) electrons from gold to CeO_2_ (in an opposite direction than that under UV irradiation).

Probably, the hottest topic in semiconductor photocatalysis is nowadays the formation of S-scheme heterojunctions (previously known as Z-scheme [12]), which is caused by their favorable photocatalytic performance, i.e., light absorption at broad solar spectrum (from UV to near IR) and excellent redox properties, resulting from the co-existence of two different components (with high oxidation and reduction power). In the case of photocatalytic gels, different composites have been proposed (mostly S-scheme junctions), and many of them consist of C_3_N_4_ and graphene-based materials, such as graphene (G), graphene oxide (GO), and reduced graphene oxide (rGO). In the case of C_3_N_4_, in addition to the composite, reduction ability is increased, and the vis response is guaranteed, whereas graphene-based materials participate in an efficient electron migration. For example, BiOBr/RGO, C_3_N_4_/TiO_2_/SiO_2_/PAN, CdS/C_3_N_4_/G, Ag/AgBr/BiVO_4_/G, C_3_N_4_/Fe_2_O_3_/G, BiVO_4_/CeVO_4_/RGO, BiOI/C_3_N_4_/G, C_3_N_4_/TiO_2_/ZnIn_2_S_4_, CaBi_2_O_4_/G, and WS_2_/C_3_N_4_/GO have been prepared and used for various photocatalytic reactions, as shortly presented below. It should be pointed out that, usually, only one material is in the form of a gel, whereas other components are just deposited on it or/and in the porosity of the gel structure. However, uniform composite gels have also been synthesized. For example, BiOBr/rGO cryogel was prepared through a hydrothermal method using BiOBr (also pre-synthesized hydrothermally) and GO precursors, and dopamine as a reducing agent for GO; then, freeze-drying was used to remove solvents from the hydrogel [116]. It has been found that BiOBr/rGO cryogel exhibits higher activity than the reference sample (BiOBr cryogel) for photocatalytic degradation of the two dyes and phenol under UV irradiation (though vis activity has been claimed for irradiation with an Xe lamp equipped with a 360 nm-cut-off filter), possibly due to a faster migration of photogenerated electrons via the rGO network.

**Figure 10:**
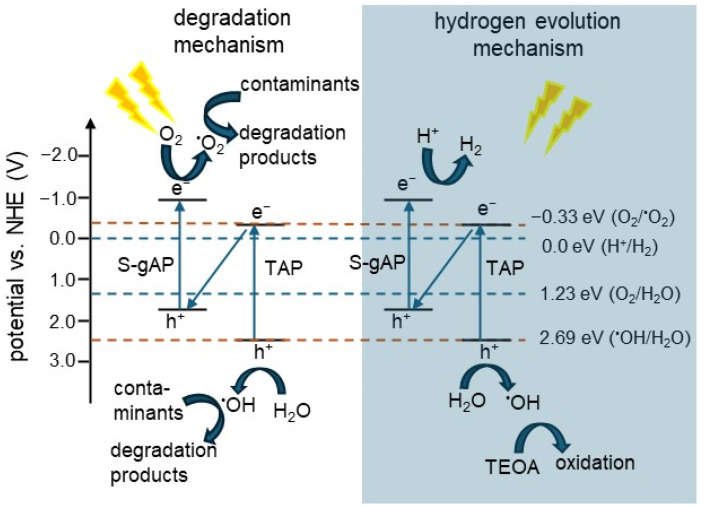
The schematic illustration of possible S-scheme mechanisms for charge separation and migration during photocatalytic degradation of organic pollutant (**left**) and hydrogen evolution (**right**) over a quaternary photocatalyst; S-gAP: S-C_3_N_4_/SiO_2_ and TAP: TiO_2_/SiO_2_/PAN. Based on Reference [81]. Copyright (2022) Elsevier.

The S-scheme example has been presented for a quaternary photocatalyst composed of sulfur-doped C_3_N_4_ (S-C_3_N_4_), titania, silica, and PAN [81]. In this composite, the two first components are photocatalytic materials, whereas silica and PAN are used for morphology control—to form a good-quality cryogel structure (solving a brittleness problem of nanofibers—a gel precursor). Based on photoelectrochemical and photocatalytic activity studies, it has been proposed that enhanced activity for both oxidation (degradation of dyes and antibiotics) and reduction (hydrogen evolution) reactions is caused by the S-scheme mechanism, as presented in Figure 10.

Another interesting approach has been proposed for a ternary photocatalyst composed of C_3_N_4_, CdS, and graphene cryogel [117]. In this composite, the S-scheme mechanism between C_3_N_4_ and CdS has been enhanced by an efficient electron migration via a graphene 3D network. Similarly, graphene cryogel has been proposed for an activity enhancement due to an efficient electron transfer (its high conductivity), extended light lifetime, and a large specific surface area, in the case of the quaternary photocatalyst: C_3_N_4_/TiO_2_/ZnIn_2_S_4_/graphene [118]. Here, double S-scheme junctions (i) g-C_3_N_4_/TiO_2_ and (ii) TiO_2_/ZnIn_2_S_4_ have been proposed as the main reason for high photocatalytic activity. Moreover, it has been claimed that the 3D network structure of cryogel (besides extending the light-harvesting ability) might also accelerate reagents’ adsorption, i.e., forming active reaction sites, which in turn further improves the photocatalytic efficiency. There are many similar studies showing that graphene-based aerogels/cryogels cause activity enhancement for S-scheme junctions due to an enhancement of electron transfer between semiconductors, efficient light-harvesting ability, and enlarged specific surface area, such as g-C_3_N_4_/α-Fe_2_O_3_/graphene cryogel with claimed vis photocatalytic activity (but tested only for dyes’ decomposition) [119], Ag/AgBr/BiVO_4_/graphene cryogel [120], CeVO_4_/BiVO_4_/RGO cryogel [121], and BiOI/g-C_3_N_4_/graphene cryogel [122].

Additionally, graphene-based composite cryogels/aerogels containing only one semiconductor have also been designed. In these materials, graphene works as a support for semiconductors to form 3D photocatalytic structures and enhances the charge carriers’ separation through the fast transfer of electrons via a graphitic network. For example, Lv et al. have proposed CaBi_2_O_4_/graphene cryogel for the degradation of dyes and antibiotics under sunlight and solar-simulated light [123], whereas Shafi et al. have developed WS_2_ nanosheets on the interpenetrating channels of nitrogen (N)-doped graphene cryogel for caffeine degradation [124]. Moreover, it has been proposed that gels, due to floating properties and thus existing at the air–water interface, might utilize incident light efficiently for wastewater treatment and water purification (as discussed in the next section).

The summarized data for the photocatalytic application of gel-based photocatalysts are presented in Table 5. Although the high performance of various photocatalysts has been claimed, especially under vis irradiation, it should be underlined that experiments performed under vis irradiation for dyes as testing molecules could not prove the vis response because of dye sensitization, commonly used in well-known dye-sensitized solar cells [125,126,127].

### 3.3. Other Applications of Gels in Photocatalysis

Interestingly, gels have not been only used in photocatalysis as an active part—photocatalyst, but also their unique properties have been utilized for other purposes, e.g., as a support for photocatalysts, an encapsulating layer, a micromotor, and a floating agent, as presented below.

#### 3.3.1. Gels as a Support for Photocatalysts

It is well known that the most active photocatalytic materials are characterized by a large specific surface area, which usually correlates well with very fine particles of materials. Accordingly, the separation of fine photocatalysts’ NPs after reaction and their recycling relates to the high costs of ultrafiltration or other expensive separation techniques. It should also be mentioned that possible aggregation of fine particles during photocatalytic reaction could result in undesirable photocatalyst precipitation, whereas efficient stirring causes an increase in operation costs. Therefore, immobilization or packing of photocatalysts on/in various supports is commonly applied [128], such as (i) on photoreactor walls [129], (ii) on/in elements of the irradiation system (e.g., on the surface of a glass tube around a UV lamp [130], on the external surface of a glass tube with transmitted UV light by an optical fiber bundle [131], on the surface of lamps (thus lamps are named as “reactive lamps”) [132], on the surface of optical fibers coupled with LED [133], packed in a concentric Pyrex glass tube placed around a fluorescent lamp [134]), and (iii) on additional elements (e.g., glass beads [129], mesh [135], membrane [136,137,138], plates [139,140,141,142], cloth [135,143], foils [139]) packed in a photoreactor.

Additionally, various materials, mostly in the form of particles (of larger size than photocatalysts), are used as a photocatalyst support. For example, silica particles [144], silica gel [145], molecular sieves [146], activated carbon [147], zeolites [146], glass microspheres recovered from fly ashes [148], sand [149], and Raschig rings [150]. It should be noted that a support could be inert or active, e.g., participating in charge carriers’ transfer, pollutant adsorption, light harvesting, etc. [128]. Moreover, even an inert support might influence photocatalytic performance, e.g., a decrease in activity due to (i) mass transfer limitations [129], (ii) surface area decrease [139,151], (iii) loss of reactants’ adsorption [135,140,146], (iv) limitations in charge carriers’ migration and lifetimes [152], and (v) fast sedimentation of granulated photocatalyst [153]. Obviously, gels (hydrogels and their dried forms) have also been applied as a photocatalyst support, especially because of their large specific surface area, as presented in Table 6.

**Figure 11:**
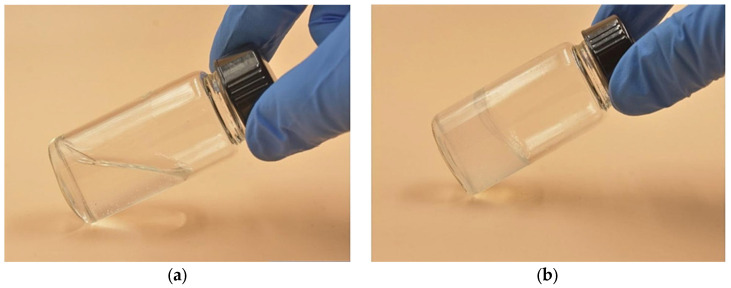
The photographs of two cellulose-based solutions kept at room temperature for 24 h after their preparation: (**a**) ZnO/cellulose; (**b**) ZnO/SiO_2_/cellulose—showing acceleration of gelation by silica. Reprinted with permission from Reference [157]. Copyright (2022) Elsevier.

Firstly, hydrogels have attracted significant interest due to their transparency, enabling efficient light penetration [154,155]. Additionally, the swelling ability of hydrogel enables penetration of pollutants/reactants inside the hydrogel structure, allowing efficient reactions with an embedded photocatalyst [156]. One of the most common hydrogels used as a photocatalyst support is composed of cellulose. For example, a cellulose-based hydrogel composite with zinc oxide/silica was fabricated by in situ synthesis [157]. It has been found that though fabrication of cellulose hydrogels could be carried out efficiently at low-temperature heating (60 °C), the introduction of silica accelerates the gelation, and thus, hydrogel might be formed even at room temperature, as shown in Figure 11. Moreover, it has been proposed that silica acts not only as a cross-linking agent (enhancing the mechanical strength and stability of hydrogel) but might also enhance the photocatalytic activity of ZnO via transferring the charge carriers through its surface states.

Alginate is another compound that could be used in the form of hydrogel. For example, in the case of g-C_3_N_4_ photocatalyst modified with platinum, alginate hydrogel has been suggested as a good support to avoid aggregation/precipitation, continuous stirring, and not environment-friendly recycling [158]. Interestingly, another important function of hydrogel has been suggested, i.e., as a water storage/reservoir, which is quite important for some reactions, especially those in which water is the main reactant, such as water splitting. The use of alginate-based hydrogel has also been proposed by Zhang et al., who synthesized iron(III)–alginate hydrogel beads and applied them for simultaneous redox conversion, i.e., reduction of chromate (VI) and oxidation of arsenite (III) [159]. Another natural material—a rubber (maleated liquid natural rubber)—has also been used as a support for the bismuth ferrite (BiFeO_3_) photocatalyst for the removal of MB dye (adsorption and degradation) under natural sunlight and simulated solar light (UV/vis) [160]. However, a significant decrease in performance has been observed, i.e., a drop in MB removal efficiency from 99.64% to 32.53% in the first and the fifth cycles, respectively, reaching the maximum capacity of dye adsorption. Moreover, the morphology of hydrogel has also been changed, i.e., the structure has been defaced, and thus, the reusability of this material is only four cycles.

Interestingly, the research on hydrogels is not only limited to their chemical composition but also their different morphology has been investigated, including beads [159], seagrass (free-standing 2D nanoassemblies) [161], and sheets [155].

Furthermore, a solid form of gel has been used as a support for photocatalysts. For example, cellulose-based cryogels have been proposed as a support for ZnO/AgBr photocatalyst modified with carbon dots (CDs) for photocatalytic reduction of Cr(VI) [162]. Interestingly, a dual function of CDs has been proposed, i.e., enhancing adsorption of Cr(VI) and improvement of photocatalytic activity (hindering charge carriers’ recombination). Moreover, the recycling tests have indicated good stability (>88% of the initial value after five cycles).

#### 3.3.2. Gels for Photocatalyst Encapsulating

An encapsulating layer/sphere could be considered as another kind of photocatalyst support, but when the “support” surrounds the photocatalyst, giving small capsules—core–shell structures with photocatalysts and gels as a core and a shell, respectively. Here, hydrogels have been mostly applied, especially for water remediation, because of adjustable permeability. For example, calcium alginate hydrogel (CA) encapsulating the BiOBr_0.75_I_0.25_ (BOBI) beads’ photocatalyst has been proposed for efficient removal of oxytetracycline (OTC) antibiotic from wastewater [163]. The photocatalyst was synthesized by a solvothermal method, whereas hydrogel beads were prepared through ionic gelation of sodium alginate with calcium chloride. Although CA-BOBI exhibits superior adsorption performance in comparison to the reference sample BOBI, its activity in OTC removal is slightly lower, probably due to limited light penetration inside the beads. Nevertheless, the recycling experiments for CA-BOBI have confirmed its high stability, even after the fifth cycle (only ca. 5% activity loss). It should be pointed out that the overall performance of both photocatalysts (CA-BOBI and BOBI) is rather similar, but recycling the encapsulated photocatalyst is much easier and cheaper. Moreover, the use of encapsulated materials seems much more profitable for environmental aspects due to a lower possibility of their interaction with living organisms [11].

**Figure 12:**
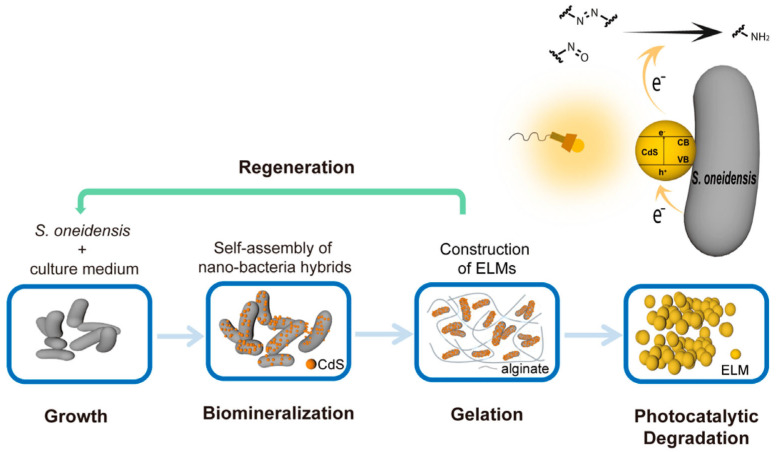
The scheme showing the construction of engineered living materials (ELMs) with encapsulated nano-bacteria hybrids: CdS NPs biomineralized by *Shewanella oneidensis* deposited on the surface of cells for self-assembly of nano-bacteria hybrids; the hybrids encapsulated in an alginate hydrogel containing nutrients to form ELMs. Nano-bacteria hybrids in the ELMs with possible application to remove contaminants via photocatalytic reductive degradation (top right). Reprinted from Reference [164]. Copyright under the terms of the Creative Commons Attribution License.

A very interesting approach has been proposed for a photocatalyst hybrid, CdS deposited on living Gram-negative bacteria *Shewanella oneidensis*, encapsulated by alginate hydrogel, and named Engineered Living Material (ELM), as shown in Figure 12 [164]. It has been claimed that encapsulation improves the resistance of material to environmental stress, preserving the photocatalytic properties of nano-bacteria hybrids. Moreover, ELM might enhance the viability of nano-bacteria hybrids, as non-encapsulated ones cannot survive for more than three days, whereas encapsulated nano-bacteria hybrids exhibit photocatalytic degradation capacity (and viability) for more than four weeks. It has been concluded that the unique properties of ELM (recyclability, stability, and re-generativity) make them an attractive material for real wastewater treatment.

**Figure 13:**
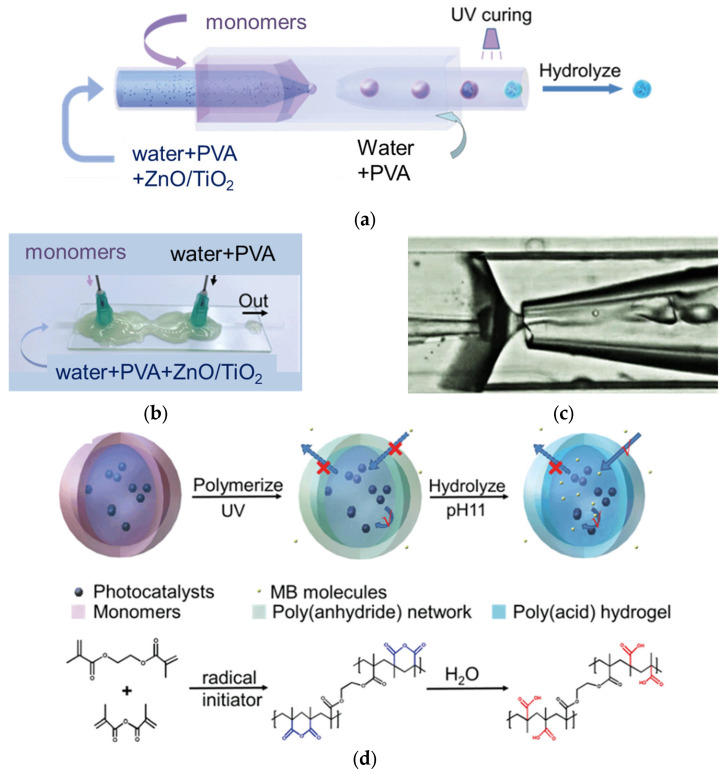
The schematic drawings with respective images of the preparation of hydrogel microcapsules with an aqueous core containing photocatalytic nanoparticles: (**a**) schematic graphs; (**b**) optical image; (**c**) optical microscopy image of the capsules generation using glass capillary microfluidic device; (**d**) photo-polymerization of methacrylic anhydride and ethylene glycol dimethacrylate leading to a poly(anhydride) network in the shell, which after hydrolysis forms poly(acid) hydrogel microcapsules. Adapted from Reference [165]. Copyright (2020) RSC.

Liu et al. have proposed an interesting synthesis method for the preparation of photocatalysts encapsulated by a poly(methacrylic acid) hydrogel shell through a glass capillary-based microfluid technique, as shown in Figure 13 [165]. First, three mixtures were prepared, i.e., (i) photocatalyst (ZnO or TiO_2_), polyvinyl alcohol (PVA), and water as an “inner phase”, (ii) PVA and water as an “outer phase”, and (iii) ethylene glycol dimethacrylate (EGDMA) and methacrylic anhydride (MAAn) as a “middle phase”. The outer phase, the inner phase, and the middle phase were injected into the respective inlets, and the double emulsion drops were formed between the inlet and the outlet capillary tip (Figure 13a,c). Next, the drops were UV-irradiated, and then one-day hydrolysis was carried out (drops immersed in KOH solution). It has been proven that these capsules can work as efficient chemical microreactors for the adsorption and degradation of pollutants.

#### 3.3.3. Gels as Micro/NanoMotors/Microrobots

Micro/nanomotors (MNMs) are becoming more and more popular, and the modeling of their properties (sizes, shapes, motion, and functions) has been intensively investigated for various applications, especially in biomedical and environmental fields [166,167,168,169,170]. Accordingly, gels have also been proposed as a support for various active materials (catalysts, photocatalysts, magnetic particles, etc.), forming functionated MNMs. For example, Lin et al. prepared three types of micromotors by capping hydrogel microspheres with functional NPs [171]. Microspheres of 20–50 µm diameter were prepared in droplet microfluidics based on hydrogel polymerization. Then, through solidification of the hydrogel layer onto microspheres, NPs of MnO_2_ (for catalytic evolution of O_2_), TiO_2_ (for photocatalytic evolution of O_2_), and Fe_3_O_4_ (for magnetic guidance) were deposited on the surface of microspheres. Indeed, photocatalytic-active MNMs (with titania), in the presence of H_2_O_2_ and under UV irradiation, propel microspheres because of photogenerated O_2_ bubbles. Similarly, oxygen bubbles, generated by the photocatalytic decomposition of hydrogen peroxide under irradiation, drove microswimmers [172]. In that study, a 3D printer was used to prepare various shapes of micromotors, such as claw-like, hook-like, fish-like, and helical-like. The correlation between morphology and movement speed has been observed, as presented in Table 6.

A very interesting study has been proposed by Maria-Homingos et al. on a simple one-pot synthesis of rod-like chitosan (CHI) hydrogel containing magnetic (Fe_3_O_4_) and photocatalytic (ZnO) NPs [173]. It has been found that these soft microrobots could efficiently decompose parathion pesticide under UV irradiation, probably due to the synergistic effect of photocatalytic activity (ZnO), solution intermixing by microrobots’ motion (Fe_3_O_4_), and efficient pesticide adsorption on CHI. Moreover, the magnetic properties of microrobots allow photocatalyst reuse. Although photodegradation efficiency decreases after five cycles (ca. 60% initial activity), the used materials are biocompatible and/or biodegradable and, thus, environmentally friendly. They are ideal for in situ decomposition of pesticides, microplastics, and other pollutants into harmless products [174].

Hydrogel microrobots overcome problems related to side effects against normal cells caused by nanostructured photocatalysts used in anti-cancer therapy. This approach could avoid the toxicity of metal-based photocatalytic anti-cancer agents and might cause a selective activation of drugs at specified tumor sites. Many hydrogels have excellent biocompatibility, biodegradability, and low toxicity, which makes them an ideal material for soft microrobots with excellent flexibility and adaptation to a wide range of shapes and the ability to cross difficult-to-reach regions in a human body (e.g., the blood–brain barrier). The porous hydrogel layer enables modulation of MNMs powered by light or a magnetic field and, thus, precise motion control and localization. Stimuli-responsive synthetic hydrogels like poly(N-isopropylacrylamide), polyacryl acid (PAA), and their copolymers have been used for the formation of robots’ systems operating in human body environmental conditions [175]. For example, an early-stage photodynamic cancer therapy under vis irradiation was proposed by Galata et al. [176]. Doped titania NPs were embedded in an interpenetrating network of poly(N-Isopropylacrylamide-co-polyacrylicacid)–pNipam-co-PAA. Then, the obtained pNipam-co-PAA/(co)doped-TiO_2_ particles were in vitro tested against two breast cancer lines: highly invasive MDA-MB-231 (human breast adenocarcinoma) and MCF-7 (Michigan Cancer Foundation) with low metastatic potential, under vis illumination and in dark conditions. The polymeric microgel efficiently releases photocatalyst NPs upon or very close to the cancer cell membranes, while the non-embedded NPs disperse everywhere in the cell culture medium. The photoactivation under vis irradiation of composites leads to selective inhibition of proliferation of MDA-MB-231 cells by generated ROS (reactive oxygen species), whereas no significant effects on cell proliferation were observed for MCF-7 (non-metastatic) cancer cells and non-embedded titania NPs [176].

#### 3.3.4. Gels as Floating Agents

Although photocatalyst immobilization has been commonly applied for its efficient separation after reaction, it could also result in the creation of new problems with insufficient light penetration, especially when a photocatalyst is deposited on large/heavy supports, placed often on the bottom of the photoreactor (or water/wastewater tanks/reservoirs). Accordingly, a floating photocatalyst might be a good solution.

Indeed, a floating photocatalyst has been designed by Lee et al., who propose the connection of hydrophilic and hydrophobic properties in one composite (as a Janus-like structure), i.e., for a support and a photocatalyst, respectively, which results in achieving a floating feature [177]. Therefore, hydrophilic polyurethane and polyethylene glycol-based aerogel were used for both parts, but photocatalyst (Pt- or Cu-modified titania) and hydrophobic silica aerogel were introduced only to the upper part, which allowed photocatalyst floating (only the hydrophilic down part was immersed in water). Accordingly, this structure is known as a traditional “half-floating” one. Interestingly, the comparison of this photocatalyst with sunken material indicates that the floating photocatalyst exhibits twice higher efficiency of hydrogen evolution, probably due to an efficient light penetration, but also better gas separation, and thus hindered back-oxidation of H_2_.

**Figure 14:**
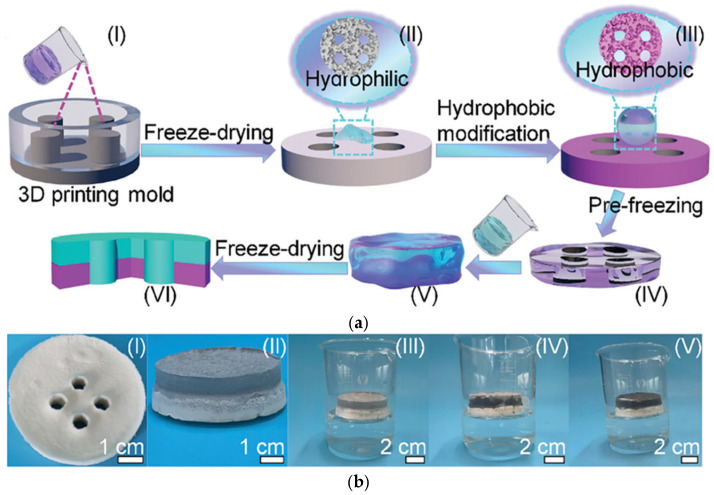
The schematic drawings of MTSJA synthesis with respective photographs: (**a**) the synthesis scheme of BBLA part ((I)–(III)) and ULLA part ((IV)–(VI)); (**b**) photographs of BBLA part (I), MTSJA (II), and self-floatable MTSJA on the water surface ((III)–(V)). Reproduced from Reference [178]. Copyright under the terms of the Creative Commons Attribution License.

An opposite structure, i.e., composed of a hydrophilic top and a hydrophobic bottom (and thus fully floatable), has been proposed by Li et al. [178]. The authors have been inspired by an ancient Chinese craft, i.e., mortise-and-tenon architecture. Accordingly, the mortise-and-tenon structural Janus aerogel (MTSJA) was synthesized with a hydrophilic tubular-like structure with sticks (mortise part, ULLA) and a hydrophobic bottom part with holes (tenon part, BBLA), as shown in Figure 14.

Another form of a floating material has been proposed by Dalponte et al., who designed floating beads [179]. The low-density alginate-based titania photocatalyst was synthesized by ionotropic gelation with gas-forming agents (CaCO_3_, NaHCO_3_). Interestingly, the floating photocatalyst (without any stirring) exhibits only slightly lower and significantly larger (two times) photocatalytic activity (analyzed for tetracycline degradation under UV) than that by suspended P25, tested with stirring and without stirring, respectively. Additionally, good stability of floating beads has been noticed, especially towards the degradation of tetracycline (similar or even slightly larger effect after the 7th cycle), but a decrease (3×) in mineralization (total organic carbon (TOC) removal) indicates that biopolymers (alginate) are susceptible to degradation under long-term UV irradiation, due to cleavage of glycosidic covalent bonds. Accordingly, an addition of a more stable component, i.e., Brazilian bentonite (BB), has been proposed [180]. Indeed, the floating titania/alginate/BB photocatalyst beads exhibit very good stability during recycling for both the degradation of chemical compounds and TOC removal.

**Table 6:** Exemplary data for other applications of gels.

Gel Form	Gel Role	Photocatalyst	Activity Tests			Ref.
			Irradiation	System	Effect *	
TOCNs/PAM h.	photocatalyst support and water storage	TiO_2_	UV/vis	d. MO	97%, 90 min	[154]
transparent wood (lignin) h.		Bi-N-CDs /BiOBr	vis	d. RhB	93%, 300 min	[155]
cellulose h.		ZnO/SiO_2_	UV/vis	d. MB	95%, 120 min	[157]
cellulose c.		ZnO/AgBr/ CDs	UV/vis	r. Cr(VI)	96%, 120 min	[162]
MLN rubber h.		BiFeO_3_	sunlight UV/vis UV/vis	a/d. MB	60%, 180 min 99%, 180 min 5-cycle: 33%	[160]
alginate h. beads		Fe^3+^	UV/vis	r. Cr(VI) o. Ar(III)	100%, 150 min	[159]
alginate h.		Pt/gC_3_N_4_	UV AS/mon AS/mon	ev. H_2_	7437 μmol g^−1^ h^−1^ (H_2_) Φ_app(420 nm)_ = 1.88% Φ_app(550 nm)_ = 0.21%	[158]
CA h. beads	encapsulation	BiOBr_0.75_I_0.25_	vis	d. OTC	91%, 60 min	[163]
alginate h.		*S. oneidensis*-CdS	UV/vis	r. TB r. NGB	68%, 24 h 60%, 24 h	[164]
PMAA h.		ZnO TiO_2_	UV	a/d. MB	100%, 20 min 100%, 20 min	[165]
PEGDA h.	micromotors	TiO_2_	405 nm	movement	CL: 300 μm s^−1^ HL: 170 μm s^−1^ HL: 100 μm s^−1^ FL: 100 μm s^−1^	[172]
chitosan h.		ZnO	UV	d. parathion	75%, 30 min	[173]
HPU/PPG/SiO_2_ a.	floating agent	Pt/TiO_2_ Cu/TiO_2_	sunlight	ev. H_2_	163 mmol h^−1^ (H_2_) 79 mmol h^−1^ (H_2_)	[177]
PVA-co-PE c.		SNCN@GQD/CdS	vis	r. H_2_O r. CO_2_ r. CO_2_	19 μmol g^−1^ h^−1^ (H_2_) 9.5 μmol g^−1^ h^−1^ (CO) 3 μmol g^−1^ h^−1^ (CH_4_)	[178]
alginate		TiO_2_	UV	d. tetrazine	89%, 180 min	[179]

* rounded to the nearest integer (when possible), 5-cycle—the effect after 5th repetition, a.—aerogel, a/d.—adsorption and degradation, AS/mon—action spectra, i.e., with monochromatic irradiation; CA—calcium alginate, CDs—carbon dots, c.—cryogel, d.—degradation, ev. H_2—_evolution of hydrogen, GQD—rGO quantum dot, h.—hydrogel, HPU—hydrophilic polyurethane, MB—Methylene Blue, MLN—maleated liquid natural, MO—Methyl Orange, MTSJA—mortise-and-tenon structural Janus aerogel, NGB—naphthol green B, o.—oxidation, OTC—oxytetracycline, *S. oneidensis*—*Shewanella oneidensis*, PEGDS—polyethylene glycol diacrylate, PMAA—poly(methacrylic acid), PPG—poly(propylene glycol), PVA-co-PE—polyvinyl alcohol and polyethylene, SNCN—S─N co-coped g-C_3_N_4_, r.—reduction, RhB—rhodamine B, TB—trypan blue, TEMPO/TOCNs/PAM—2,2,6,6-tetramethylpiperidine-1-oxyl (TEMPO)-oxidized chitin nanofibers (TOCNs), which were further incorporated into the polyacrylamide (PAM), Φ_app(λ)_—apparent quantum yield.

## 4. Conclusions

Photocatalysis is considered a possible future method for solving the most urgent of humanity’s problems, i.e., water, environment, and energy. Accordingly, it is believed that solar-based technologies with active and stable photocatalysts could be broadly used, especially in the world regions with high sunlight exposition, and thus areas with the largest and the most reasonable needs (Africa and a large part of Asia). Although various photocatalytic materials have already been designed, synthesized, characterized, and tested, the real applications are very limited (e.g., photocatalytic paints and coatings with self-cleaning and antifogging properties). The two most important problems are connected to recyclability and stability. Accordingly, the application of photocatalysts in the form of gels, preventing agglomeration, could be a good solution. Indeed, easy recycling has been reported in most cases, as clearly presented in this review, but the stability issues have not been fully addressed. Although some reports have already presented the stability study during recycling, the contrary results could be found, i.e., a low stability (due to the destruction of the gel structure) or a high stability, but only for specially stabilized structures. Accordingly, this problem should be investigated and solved in future studies.

Interestingly, the reported photocatalytic activity in many cases is similar to that of suspended particulate photocatalysts (even highly active P25), resulting probably from high specific surface area and efficient light penetration (e.g., efficient light harvesting ability due to a porous structure and a floating feature). Unfortunately, many activity tests have been performed for discoloration of dyes under vis irradiation, which is improper because of the sensitization mechanism. Therefore, even though vis activity is claimed, further experiments should be performed to confirm photocatalytic activity at a broad solar spectrum. Moreover, in some cases, even irradiation used for vis experiments has not been proper, e.g., xenon lamps (with UV/vis emission) and a wrong cut-off filter (λ > 360 nm).

There are also some minor problems with nomenclature differently used in various reports. Commonly, an “aerogel” term is used for all solid gels, independently of the used technique for their preparation, but most materials have been dried by freeze-drying, and thus, a “cryogel” term is more proper.

Despite some problems with reported data, it might be concluded that gel photocatalysts are highly promising materials for broad environmental applications when the stability issue is successfully solved.

## Figures and Tables

**Table 1:** The comparison of titania aerogels’ synthesis methods.

Method	Synthesis Process	Advantages	Disadvantages
sol-gel	hydrolysis and polycondensation/aging	simplicity, low cost	supercritical drying, amorphic nature of aerogels (calcination need)
self-assembly	surface polymerization of crystalline TiO_2_ NPs	microporous and crystalline products without calcination	forming inability of long chains of particles, mostly one-dimensional chains
gas-phase	evaporation and decomposition of Ti-containing aerosol to form titania	high purity, crystalline products	controllability problems, special gas-phase equipment

NPs—nanoparticles.

**Table 2:** Exemplary data of titania aerogels with representative photocatalytic activity.

Sample	Synthesis Method	BET * /m^2^ g^−1^	Photocatalytic Activity Tests			Ref.
			Irradiation	System	Effect *	
TiO_2_	acid-catalyzed sol-gel	691	UV	d. salicylic acid	~60%, 450 min	[58]
TiO_2_	sol-gel with freeze-drying	16	simulated solar light	d. MB d. caffeine d. *E. coli* d. *E. faecalis*	K: 0.265 min^−1^ K: 0.054 min^−1^ inactivation inactivation	[72]
TiO_2_	self-assembly	302	vis	d. RhB	~100%, 30 min	[67]
TiO_2_-WO_x_-Au nanowires	self-assembly	473	UV	d. MB	60%, 120 min	[73]

d.—degradation, RhB—Rhodamine B, MB—Methylene Blue, *E. coli—Escherichia coli*, *E. faecalis—Enterococcus faecalis*, K—reaction rate constant, * rounded to the nearest integer (when possible).

**Table 3:** Examples of titania–silica aerogel photocatalysts.

Sample	Synthesis Method	BET * /m^2^ g^−1^	Photocatalytic Activity Tests			Ref.
			Irradiation	System	Effect *	
TiO_2_/SiO_2_ different TiO_2_ content	SG titania adsorption	750	UV	d. RhB	~83%, 200 min	[74]
TiO_2_/SiO_2_ different Si:Ti ratio	SG solvent exchange	726	UV	d. MB	96%, 70 min	[75]
	SG air drying, calcination	358	UV	d. pyridine	~83%, 480 min	[76]
hydrophobic TiO_2_/SiO_2_	In situ polymerization SG; autoclave drying	252	UV	d. MB	87%, 120 min	[77]
mesoporous TiO_2_/SiO_2_	SG, solvent evaporation, supcrit. drying	840	UV	c. TCE in gas-phase	19%	[78]
TiO_2_/SiO_2_ monoliths	evaporation-induced self-assembly	814	UV	d. 4-CP	86%, 60 min	[79]

d.—degradation, c.—conversion, MB—Methylene Blue, TCE—trichloroethylene, 4-CP—4-chlorophenol, * rounded to the nearest integer (when possible), SG—sol-gel, supcrit.—supercritical.

**Table 5:** Exemplary data for gel-based photocatalysts.

Sample	Synthesis Method	BET * /m^2^ g^−1^	Photocatalytic Activity Tests			Ref.
			Irradiation	System	Effect *	
CdS	sol-gel	187	vis	d. MB d. MO	80%, 50 min 90%, 150 min	[96]
CdS QDs	assembly	180	vis	CO_2_ reduction	296 μmol g^−1^ h^−1^ (CH_4_)	[101]
CdSe QDs	assembly	276	vis		30 μmol g^−1^ h^−1^ (CO)	[102]
S-gC_3_N_4_/ TiO_2_/SiO_2_/PAN	assembly	309	sim. solar	d. MB d. RhB d. MO d. TC ev. H_2_	99%, 15 min 96%, 30 min 91%, 40 min 84%, 40 min 806 μmol g^−1^ h^−1^ (H_2_)	[81]
CdS/gC_3_N_4_/G	sol-gel	153	vis	ev. H_2_ d. RhB	86 μmol g^−1^ h^−1^ (H_2_) K: 0.049 min^−1^	[117]
BiVO_4_/RGO/CeVO_4_	sol-gel	44	vis	d. TC	K: 0.045 min^−1^	[121]
gC_3_N_4_/TiO_2_/ZnIn_2_S_4_/G	assembly	133	sim. solar	d. MO r. Cr(VI) ev. H_2_	97.5%, 30 min 98.3%, 70 min 6532 μmol g^−1^ h^−1^ (H_2_)	[118]
Ag/AgBr/BiVO_4_/G	assembly	-	sim. solar	d. MO d. *E. coli* d. *S. aureus*	K: 0.11 min^−1^ 7 × 10^9^ CFU mL^−1^ 2.9 × 10^9^ CFU mL^−1^	[120]
Au/CeO_2_	sol-gel	151	UV/vis UV/vis vis	CO_2_ reduction	7 μmol g^−1^ h^−1^ (CH_4_) 1 μmol g^−1^ h^−1^ (CO) 0.9 μmol g^−1^ h^−1^ (CH_4_)	[115]
CaBi_2_O_4_/G	assembly	-	vis	d. MB d. RhB d. MO d. TC	91%, 120 min 87%, 120 min 93%, 120 min 93%, 120 min	[123]
S-defect MoS_2_	sol-gel	-	sim. solar	d. MB	91%, 180 min	[114]
gC_3_N_4_	assembly	133	vis	ev. H_2_	30 μmol h^−1^ (H_2_)	[106]
gC_3_N_4_/α-Fe_2_O_3_/G	assembly	120	vis	d. MB	K: 0.005 min^−1^	[119]
BiOBr/RGO	sol-gel	20	UV/vis	d. MB d. RhB d. phenol	80%, 60 min 50%, 60 min 35%, 60 min	[116]
BiOI/gC_3_N_4_/G	assembly	-	vis	d. MB d. LVF	28%, 15 min 18%, 15 min	[122]
WS_2_/N-doped G	assembly	35	vis	d. caffeine	93%, 180 min	[124]

d.—degradation, ev. H_2_—evolution of hydrogen, K—reaction rate constant, LVF—levofloxacin hydrochloride, MB—Methylene Blue, MO—Methyl Orange, r.—reduction, sim. solar—simulated solar light; RhB—Rhodamine B, TC—tetracycline, * rounded to the nearest integer (when possible).

## Data Availability

No new data were created or analyzed in this study.
